# The tongue map and the spatial modulation of taste perception

**DOI:** 10.1016/j.crfs.2022.02.004

**Published:** 2022-03-18

**Authors:** Charles Spence

**Affiliations:** Crossmodal Research Laboratory, University of Oxford, United Kingdom

**Keywords:** Tongue map, Gustatory receptors, Spatial localization, Tatse perception, Multisensory interactions, Oral referal

## Abstract

There is undoubtedly a spatial component to our experience of gustatory stimulus qualities such as sweet, bitter, salty, sour, and umami, however its importance is currently unknown. Taste thresholds have been shown to differ at different locations within the oral cavity where gustatory receptors are found. However, the relationship between the stimulation of particular taste receptors and the subjective spatially-localized experience of taste qualities is uncertain. Although the existence of the so-called ‘tongue map’ has long been discredited, the psychophysical evidence clearly demonstrates significant (albeit small) differences in taste sensitivity across the tongue, soft palate, and pharynx (all sites where taste buds have been documented). Biases in the perceived localization of gustatory stimuli have also been reported, often resulting from tactile capture (i.e., a form of crossmodal, or multisensory, interaction). At the same time, varying responses to supratheshold tastants along the tongue's anterior-posterior axis have putatively been linked to the ingestion-ejection response. This narrative review highlights what is currently known concerning the spatial aspects of gustatory perception, considers how such findings might be explained, given the suggested balanced distribution of taste receptors for each basic taste quality where taste papillae are present, and suggests why knowing about such differences may be important.

## Highlights

• The existence of the tongue map has long been discredited.
• Taste receptors in the oral cavity respond to all tastes regardless of their location.
• Human psychophysical data highlights a significant spatial modulation of taste perception in the oral cavity.
• Highly-controlled studies of taste psychophysics rarely capture the full multisensory experience associated with eating and drinking.

## Graphical abstract

**Figure:**
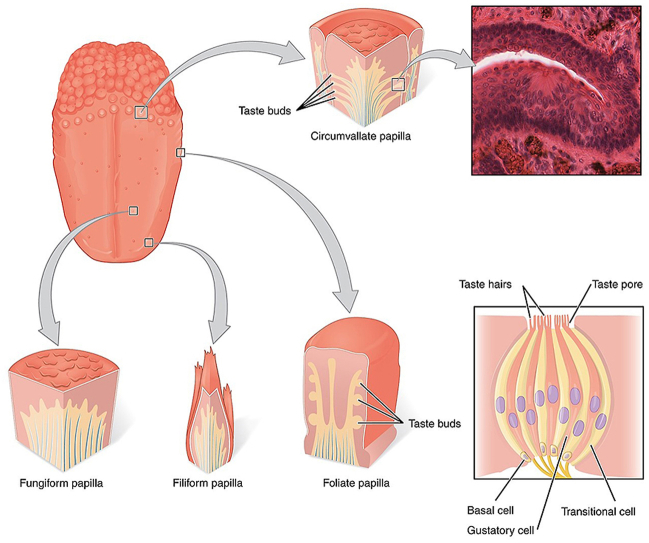

## 1 Introduction

**Fig. 1:**
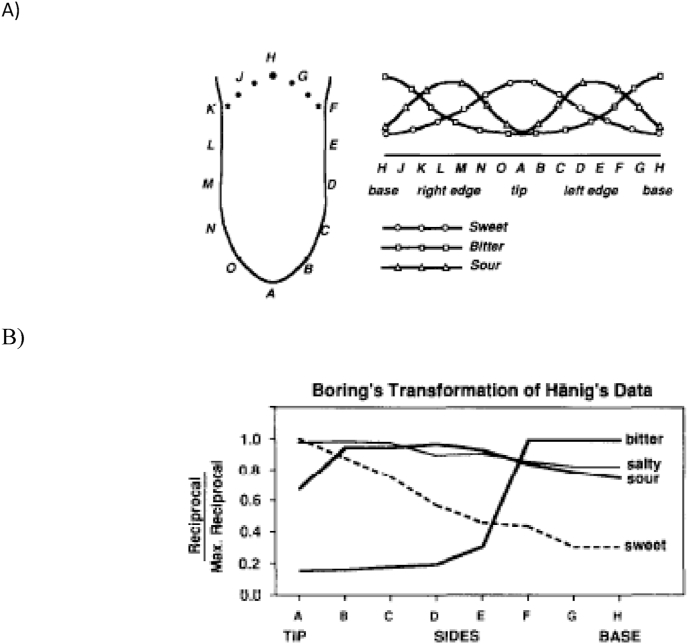
A) The sketch of the tongue on the left shows the locations at which Hanig (1901) measured taste thresholds (redrawn from Hanig, 1901; Fig. 1). The sketch on the right shows Hänig's summary of his results (redrawn from Hanig, 1901; Fig. 5). Hänig meant to show how sensitivity (the reciprocal of threshold; displayed on the ordinate) changed across the various tongue loci. Note that Hänig did not plot saltiness as it was perceived approximately equally on all loci tested on the tongue. B) Boring's transformation of Hänig's data (Boring, 1942, p. 452). For each stimulus, Boring calculated the reciprocals of Hänig's thresholds and then divided them by the maximum reciprocal. As Bartoshuk (1993, p. 23) highlights: *“On Boring's figure, there is no way to tell how meaningful the sizes of the variations are on the ordinate. Boring's graph led other authors to conclude that there was virtually no sensation as the loci where the curves showed a minimum sensation and that there was maximum sensation where the curves showed a maximum and so we have the familiar tongue maps labelled ‘sweet’ on the tip of the tongue, ‘bitter’ on the base of the tongue, etc.”* According to Bartoshuk, Boring's graph is potentially misleading as it makes small differences in sensitivity, such as between sweetness at locations A and H, when they really only represent a relatively small change in sensitivity (in this case that the threshold at H was a third of what it was at A) [Reprinted from Marshall (2013), with permission.].

During the middle decades of the 20th Century, it was widely believed that the gustatory receptors responsible for coding different basic taste properties (such as sweet, sour, bitter, and salty) were asymmetrically distributed over the surface of the tongue. According to the now discredited tongue map (see Amerine et al., 1965; Bartoshuk, 1993; Feeney and Hayes, 2014), sweet receptors were thought to be located on the front of the tongue, bitter receptors on the back, and receptors capable of detecting salt and sour tastes on the sides. The emergence of the tongue map was linked by Linda Bartoshuk (1978) to the publication of Edwin G. Boring's 1942 textbook *Sensation and perception in the history of experimental psychology,* in which the famous North American psychologist redescribed David Pauli Hanig's (1901) thesis data published in an earlier German text entitled ‘The psychophysics of taste’. In a review that appeared almost three decades ago, Bartoshuk highlighted the ambiguity inherent in Boring's (1942) replotting of Hanig's (1901) original data (see Fig. 1). Bartoshuk also suggests that Boring's text may inadvertently have been responsible for the emergence of the tongue map in articles (such as in a *Scientific American* article by Haagen-Smit, 1952) as well as in many textbooks that appeared over the following decades (see also Hammond, 2017).

One of the most striking things about the taste map is that early researchers (such as Haagen-Smit, 1952) and the public at large (or at least those writing the textbooks) could have been so wrong about the spatial qualities of taste perception for so long. As Bartoshuk (1993, p. 23) notes: *“The apparent simplicity of the tongue map has made it a popular laboratory demonstration in children's biology classes. The popularity of this laboratory demonstration is particularly amazing considering that it must fail to produce the expected results quite regularly.”* However, that being said, when high-school students were challenged to evaluate the tongue map as part of a Science Olympiad held in Arizona a few years ago, a large number of the groups did find support for the suggestion that each of the basic taste qualities is experienced somewhat differently on different parts of the tongue (see Marshall, 2013).

Contemporary research has revealed that the taste receptors capable of detecting each of the five basic tastes (bitter, sweet, salty, sour and umami) are all to be found distributed in a somewhat idiosyncratic manner across the surface of the tongue (Chandrashekar et al., 2006; cf. Harper et al., 1966). Taste receptors have also been documented at several other locations in the oral cavity, including the soft palate and larynx. According to Breslin (2013, p. R409): *“Humans taste with the edges and dorsal surface of the tongue, soft palate (the roof of the mouth toward the back of the oral cavity), and pharynx (*Fig. 1*) (**Breslin and Huang, 2006**). These tissues comprise the gustatory epithelia. We do not taste with our lips, the underside of our tongue, our hard palate (behind our upper incisors), or the inside of our cheeks”*. To demonstrate the asymmetric nature of taste perception, Bartoshuk (1993, p. 22) suggests putting: *“salt crystals on the moistened finger tip and move the crystals backward on the palate. At the margin of the hard and soft palates (where the bone under the palate ends), the salt will suddenly produce a taste.”*

The growing neuroscience understanding of the physiological transduction of, and receptor types (i.e., taste-receptor cells) involved in gustation (e.g., Dutta Banik et al., 2020; Maruyama et al., 2012; Simon et al., 2006; Tordoff et al., 2012; Wu et al., 2015; Yarmolinsky et al., 2009) might lead one to believe that taste qualities are experienced in the same way regardless of where on the tongue stimulation happens to be presented. At the same time, however, a broad array of taste psychophysics research published over the last half century or so has highlighted that sensory-discriminative (and possibly also hedonic) responses to taste sometime differ (significantly) depending on which part of the tongue/oral cavity is stimulated (see Sewards, 2004; Sewards and Sewards, 2002). How, then, should this seeming disparity between physiology and perception/psychology be resolved?^1^

### 1.1 What exactly was being claimed by those who support the tongue map?

The notion of the tongue map has been dismissed by the majority of contemporary writers (e.g., Bartoshuk, 1993; O'Connor, 2008). However, it is perhaps worth revisiting the idea, given a closer inspection of the literature soon highlights how its putative existence has been grounded on a number of rather different assumptions. In fact, the status of the tongue map depends on the specific claim about the spatial properties of taste that are being made. However, if instead, the claim is about the spatial distribution (i.e., segregation) of taste-receptor cells (for sweet, bitter, salty, and sour) then the emerging neuroscience has, in recent decades, demonstrated that the sensory receptors for the different taste qualities are to be found with a similar distribution across the tongue, and are certainly not neatly segregated as one interpretation of the tongue map can be taken to imply (Caicedo and Roper, 2001; Chandrashekar et al., 2006; Matsunami et al., 2000; O'Connor, 2008).

Chandrashekar et al. (2006, p. 288), seemingly adopt such a stance when they write that: *“Recent molecular and functional data have revealed that, contrary to popular belief, there is no tongue ‘map’: responsiveness to the five basic modalities — bitter, sour, sweet, salty and umami — is present in all areas of the tongue”*).^2^ Meanwhile, Yarmolinsky et al. (2009, p. 237) write that: *“Notably, taste buds from all regions of the oral cavity contain cells that respond to the five basic modalities. Thus, contrary to popular belief, there is no topographic map (i.e., a tongue map) of taste qualities on the tongue.”* Notice here the jump from physiology to taste qualities. One of the aims of this review is to highlight the sometimes uncertain relation between taste physiology and taste psychophysics/perception (Breslin, 2013), especially when it comes to ecologically-valid eating and drinking experiences.

If the claim underpinning the tongue map is taken to refer to the perceived spatial localization of taste qualities, and/or to differences in sensitivity to the various tastes on different parts of the tongue, then psychophysics is more likely to provide a meaningful answer than physiology. The latter interpretation appears to be what Bartoshuk (1993, p. 22) is getting at when she writes (of the tongue map) that: *“One of the most widespread ‘facts’ about taste concerns the distribution of sensitivity to the four basic tastes.”* Elsewhere, Bartoshuk (1978, p. 1074) has written that: “Collings reevaluated a “fact” about taste that is commonly believed: that sensitivity to sweet is greatest on the tip of the tongue, and sensitivity to bitter is greatest on the back of tongue. Collings pointed out that this “fact” is actually an exaggeration of the early work by Hanig.”

**Fig. 2:**
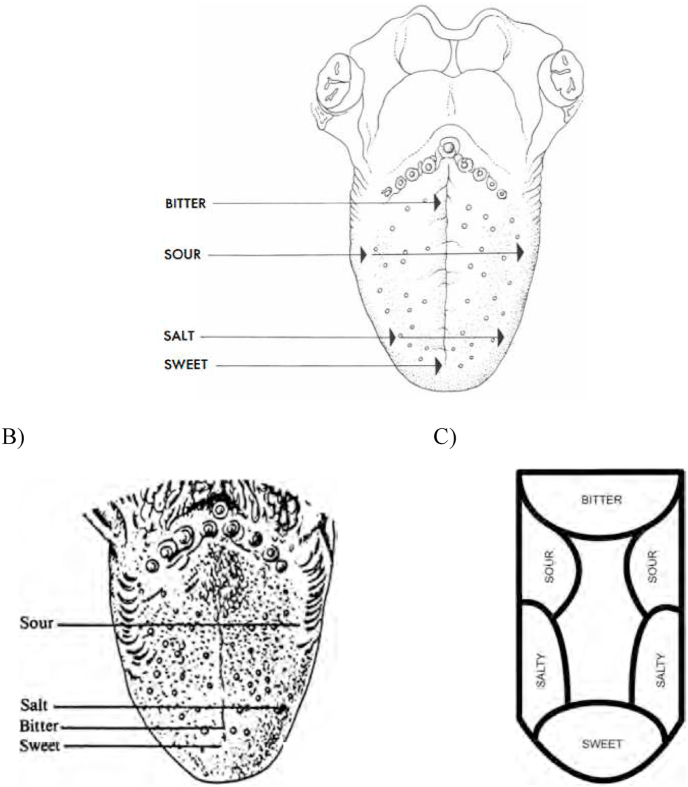
A) The localization of taste qualities on the human tongue as portrayed by Haagen-Smit (1952). According to the figure legend in the original article: *“Some areas of the tongue are more sensitive to certain tastes than others; these areas are indicated by the arrows on the top drawing”.* [Reprint from Haagen-Smit (1952, p. 29), with permission.]; B) Schiffman's (1995, p. 88) summary of variations in taste sensitivity on the human tongue: *“Approximate location on the tongue of regions of greatest taste sensitivities for the four primary taste qualities. For the bitter taste, the soft palate (not shown) is the most sensitive region”* [Reprinted from Marshall, 2013]. Notice how the location of greatest sensitivity to bitterness on the tongue has moved anterior to the preceding figure; C) A tongue map, similar to those found in textbooks and on the Web, indicating that each section of the tongue can taste only one flavor [Figure and legend reprinted from Marshall (2013).].

According to a review of taste and smell that appeared in the pages of *Scientific American,* the different parts of the human tongue are differently sensitive to each of the four most familiar basic tastes (see Fig. 2A). That is, Haagen-Smit (1952), a professor of bio-organic chemistry from the California Institute of Technology, suggested that sensitivity to bitterness is higher at the back of the tongue (i.e., posterior), sensitivity to sourness is higher on the sides of the tongue, sweetness on the front of the tongue (anterior), etc. Note, though, that while Haagen-Smit doesn't provide any references to support the claims made in his article, nor are any explicit claims made about the distribution of receptors on the tongue. As such, physiological insights might not, in-and-of-themselves necessarily be all that relevant when it comes to assessing the validity of his claim (see Fig. 2B and C for two of the other tongue maps that have appeared in the literature over the years). Here, though, the possibility should perhaps be entertained that there might be more sweet receptors at the front, and more bitter receptors at the back of the tongue, and/or that there may also be salient individual differences in the distribution of receptors. As Hoon et al. (1999, p. 541) put it more than twenty years ago now: “Circumvallate papillae are found at the very back of the tongue, contain hundreds (mice) to thousands (human) of taste buds, and are particularly sensitive to bitter substances … Fungiform papillae contain a single or a few taste buds, are at the front of the tongue, and are thought to mediate much of the sweet taste modality.”

### 1.2 Outline

In this narrative historical review, I wish to revisit the question of the spatial modulation of taste perception. I start by reviewing the literature describing the distribution of taste buds in the oral cavity (Section 2). One possible account for the anterior-posterior differences in taste perception that have been documented, based on a difference in the innervation of the taste receptor cells, is discussed. In Section 3, the available psychophysical evidence concerning the spatial modulation of taste perception is reviewed. Section 4 highlights a number of the ways in which spatial perception of taste qualities may be elicited, modulated, and/or mislocalized as a result of non-gustatory inputs. Finally, in Section 5, a number of conclusions are drawn. A distinction is also highlightedbetween the aims of traditional taste psychophysics and the contemporary desire to understand real-world multisensory flavour experiences when foods are actively masticated and swallowed, rather than when applied in a highly-controlled manner to the passive tongue of participants.

## 2 On the spatial distribution of taste buds in the oral cavity

While the majority of the taste buds are located on the surface of the tongue (Breslin, 2013; Breslin and Huang, 2006), cells with taste receptors are actually expressed throughout the gastrointestinal tract (where they may help to regulate digestion and respiration; Finger and Kinnamon, 2011; Lu et al., 2017), and have even been found in the testes and sperm (Li, 2013; Trivedi, 2012). However, only in the oral cavity does the stimulation of the taste receptor cells give rise to a conscious sensation of taste (Breslin and Spector, 2008). Even here, though, there are some contested issues. For example, certain researchers have argued that fatty acid (or oleogustus) should be considered as a basic taste (DiPatrizio, 2014; Mattes, 2011; Nachtsheim and Schlich, 2013; Running et al., 2015). However, it has also been observed that fatty acid stimuli can sometimes be discriminated under conditions of forced choice, without necessarily being associated with any phenomenal quality (so perhaps representing a kind of blind taste; Keast and Costanzo, 2015; Keast et al., 2021). Potentially relevant here is also the insufficiency of the English language to capture the distinction between taste and flavour (see Gibson, 1966; Rozin, 1982; Spence et al., 2015; Titchener, 1909). Nevertheless, should this evidence concerning the blind taste of fat be accepted, then the divide between conscious and unconscious taste suddenly becomes a little more uncertain.

**Fig. 3:**
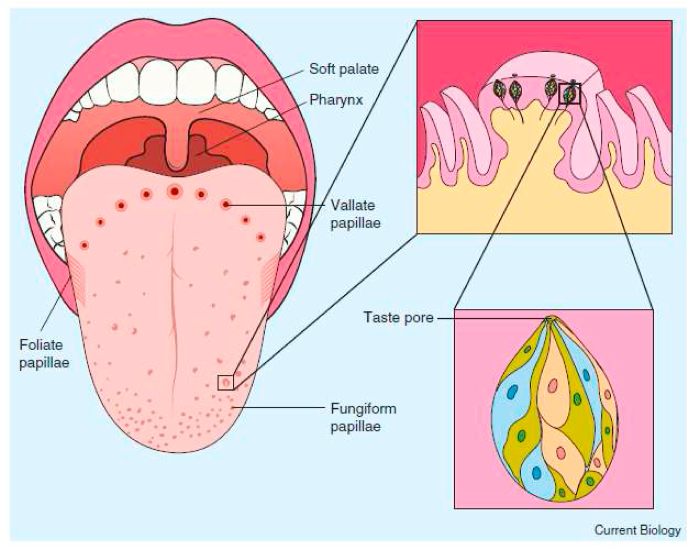
Taste papillae and taste buds of the human tongue. The human tongue contains three types of taste papillae. Vallate and foliate papillae reside on the middle and sides of the posterior 1/3 of the tongue, respectively, and contain hundreds of taste buds collectively. Circumvallate papillae comprise an arc of small ring-like structures (tiny towers surrounded by motes) in the posterior tongue. Foliate papillae are slits (leaves) in the side of posterior tongue and can appear like gills in the tongue (Pfaffmann, 1959). Fungiform papillae look like small bumps or mushrooms and are scattered in the anterior 2/3 of the tongue (though concentrated near the tip of the tongue (Jung et al., 2004; Kobayashi et al., 2004), each harbouring 0–15 taste buds (with a mean of four amongst those papillae that contain taste buds). Taste buds are also located in the flat epithelium of the soft palate (non-bony palate in front of the uvula) and pharynx (back of the throat), rather than in papillae. The first inset depicts the microscopic taste buds residing within the epithelium (outer layer) of a fungiform papilla. The small structures surrounding the fungiform papilla are called filiform papillae, which do not contain taste buds, and serve to make the surface of the tongue rough and help detect food textures. The second inset depicts a single rosette-shaped taste bud from within this fungiform papilla that contains dozens of taste receptor cells and contacts taste stimuli within the oral cavity via a small epithelial hole called a taste pore. [Reprinted from Breslin (2013, Fig. 1) with permission.].

According to Haagen-Smit (1952, p. 31): *“The sensory apparatus of taste is located chiefly on the upper surface of the tongue, at the soft palate, on the epiglottis and at the beginning of the gullet. Here lie the so-called taste buds, estimated to number around 9000.”* (see Fig. 3). The total number of taste buds has been estimated at 8000 (see Hammond, 2017). Clark and Dodge (1955) also highlighted the presence of taste buds on the tongue, palate, and nasopharynx (see Chaudhari and Roper, 2010; Witt, 2019, for excellent reviews of the cell biology and anatomy of taste). At the same time, however, as Collings (1974) has noted, the contribution of extratongue loci has not been taken into consideration in the majority of the taste psychophysics research that has been published.

Chemicals in the oral cavity are detected by taste receptor cells which are grouped together in taste buds found in epithelial specializations called papillae (Breslin, 2013; Just et al., 2005; see Fig. 3). Taste buds are onion-shaped structures of between 50 and 100 taste cells (Breslin and Spector, 2008), with Breslin (2013) putting this figure at closer to 80–100. Each taste bud has finger like-projections called microvilli that poke through an opening at the top of the taste bud called the taste pore. According to Yarmolinsky et al. (2009, p. 234): *“On the tongue, taste buds are housed within epithelial structures termed papillae, of which there are three types: (1) dozens of taste buds are distributed across the anterior surface of the tongue in fungiform papillae, (2) hundreds are located in the trenches of circumvallate papillae at the back, and (3) dozens to hundreds more localize to the sides of the tongue in foliate papillae. Many isolated taste buds are also distributed on the soft palate”.* Importantly, the gross morphology, microstructure, and innervation of these three classes of papillae differ (Sandick and Cardello, 1981). What is more, the taste buds/papillae are not distributed evenly over the surface of the tongue (see Fig. 3). The taste buds in the soft palate, pharynx, and epiglottis are not grouped in papillae (Bartoshuk, 1993).

Fungiform papillae, named after the button mushrooms that they resemble (Bartoshuk, 1993), tend to be densest on the tip and on the anterolateral margins of the tongue (Sandick and Cardello, 1981). According to Arvidson and Friberg (1980), there are an average of 1.8 taste buds per fungiform papilla, though only a little less than half of them contain taste buds (see also Arvidson, 1976; Arvidson and Friberg, 1977; Öhrwall, 1891; Plattig, 1976). The mean number of taste buds in a given fungiform papilla that contains taste buds is 4.1 (Arvidson, 1976; Arvidson and Friberg, 1980; Paran et al., 1975). Smith and Margolskee (2006) estimate there to be around 12 larger taste bud containing papillae called the circumvallate (wall-like) papillae at the back of the tongue (Sandick and Cardello, 1981, put the figure at between 8 and 12). These papillae are distributed in the shape of an inverted V, or chevron, pointing toward the throat. There are around 200–250 taste buds per circumvallate papilla (Arey et al., 1935; Mochizuki, 1937; and see Hoon et al., 1999, for a higher figure). Taste buds are also located in the foliate (leaflike) papillae, small trenches on the sides of the rear of the tongue (Breslin and Spector, 2008). According to Mochizuki (1939), there are between three and eight foliate papilla, each containing around 120 taste buds.

The three classes of taste papillae are innervated by different cranial nerves (Breslin, 2013). The front and back regions of the tongue are innervated by different cranial nerves. The anterior tongue, hosting fungiform papillae, is innervated by the lingual branch of the facial nerve, cranial nerve VII, the chorda tympani (Sandick and Cardello, 1981). The posterior third (approximately) of the tongue, incorporating both the circumvallate and foliate papillae, is innervated by the lingual-tonsilar branch of the IX^th^ cranial nerve (Breslin and Spector, 2008), known as the glossopharyngeal nerve; This nerve also carries thermal and tactile inputs (Bartoshuk, 1993). According to Simon et al. (2006), the glossopharyngeal nerve receives sensory fibres from the posterior third of the tongue, the tonsils, and the pharynx.

The soft palate, which contains taste buds on the surface of the epithelial sheet without papillary structures, is innervated by the greater superficial petrosal branch of the VII^th^ cranial nerve. (Harada et al.**,** 1997; Rollin, 1977; Yarmolinsky et al., 2009). This cranial nerve also innervates the back of the tongue (Breslin and Spector, 2008; Simon et al., 2006). Meanwhile, the taste buds posterior to the pharynx are innervated by the superior laryngeal branch of the X^th^ cranial nerve (the vagus or lingual tonsillar nerve; Breslin, 2013; Breslin and Spector, 2008).

Finger and Morita (1985) have argued that the most externally-located of the taste buds are implicated in food selection and appreciation, while those taste buds lying closest to the alimentary canal are more concerned with ingestive and protective reflexes (cf. Colvin et al., 2018). At one point, these researchers write: *“This dichotomy of function parallels the pattern of innervation of the different groups of taste buds. Taste buds lying closest to the esophagus—for example, on the palate and larynx—are innervated by branches of the vagus nerve; those lying most externally—for example, on the anterior part of the tongue” … “—are innervated by the facial nerve” … “The glossopharyngeal nerve innervates taste buds lying between these extremes.”* (Finger and Morita, 1985, p. 776). Although they were working primarily on catfish, Finger and Morita nevertheless conclude that: *“The gustatory system of vertebrates, including perhaps mammals and humans, can thus be viewed as consisting of two (or more) subsystems that mediate different behaviors.”* (Finger and Morita, 1985, p. 778). The next section reviews the human psychophysical data supporting an anterior-posterior difference in the responsiveness to certain tastants.

## 3 On the psychophysics of spatial taste

Taste psychophysics research clearly shows that sensory-discriminative thresholds for detecting each of the basic tastes in solution differs significantly depending on the location on the tongue, or elsewhere in the oral cavity, where the gustatory stimuli happen to be presented (e.g., Collings, 1974; Hanig, 1901; Nilsson, 1977, 1979; and see Table 1, for some of the earliest research on spatial differences in taste acuity). What is more, anterior-posterior differences in supratheshold taste perception have also been reported by several researchers (Feeney and Hayes, 2014; Hyde and Witherly, 1993; Sandick and Cardello, 1981). However, what is debated is the consistency and magnitude of these spatial differences. According to one article that appeared on the BBC website, *“Different areas of the tongue can taste anything, but although some regions are slightly more sensitive to certain tastes, those differences, in Steven Munger's words are “minute”.”* (Hammond, 2017). Moreover, potentially further confusing matters, Marshall (2013) has argued that individual differences in taste physiology may also lead to differing answers to the question of the nature of any spatial differences in sensitivity across the tongue. Hence, it is not just the presence (vs. absence) of any spatial modulation in taste perception that needs to be resolved, but also its consistency across individuals.

**Table 1:** Summary of Shore's (1892) early results documenting differences in the minimum percentage of four tastants in water needed to deliver a perceptible taste in himself.

	**Glycerine**	**Quinine**	**H2SO4**	**NaCl**
**Tip**	0.5	0.025	0.01	0.4
**Edge**	2.5	right .005 left .01	right .05 left .02	0.4
**Back**	1.5	0.001	0.02	0.4
**Dorsum**	No taste even in strong solutions.			

### 3.1 Sensory-discriminative threshold differences

According to the threshold studies of Shore (1892) and Hanig (1901), the greatest sensitivity to sweet tasting compounds is on the tip of the tongue, while the greatest sensitivity to bitter-tasting compounds is on the back of the tongue. Shore (1892, p. 192) writes that: *“knowing well that the power of perceiving certain tastes differs in different persons, and on different regions of the tongue of the same person, I determined that I must, at the outset, have an accurate knowledge of the perception of tastes in various parts of my own tongue.”* Concerning the taste qualities of sour and salty, Shore observed greatest sensitivity to sour-tasting compounds on the tip of the tongue, but equivalent sensitivity for salty-tasting compounds on all areas (see Table 1 for a summary of Shore's results collected on himself). In contrast, Hanig documented the greatest sensitivity for sour tasting compounds on the sides (posterior lateral margins) and for salty-tasting compounds on the tip of the tongue.

Research by Henkin and Christiansen (1967) assessed detection and recognition thresholds for the four basic tastes on the tongue, soft palate, and pharynx in 11 volunteers before and after anaesthetization of the tongue, the hard and soft palate, or both. These researchers' results revealed that sensitivity to salt and sweet tastes were greatest on the tongue whereas sensitivity for sour and bitter were highest on the palate. Henkin and Christiansen (1967, p. 316) went on to suggest that the four basic tastes are *“appreciated separately on the tongue, palate, and pharynx of man”*. It is, though, a little unclear what exactly the researchers meant to imply by the phrase ‘appreciated separately’. Others who have assessed the impact of anesthetizing the chorda tympani nerve on taste perception include Lehman (1991) and Lehman et al. (1995; see also Yanagisawa et al., 1998).

**Fig. 4:**
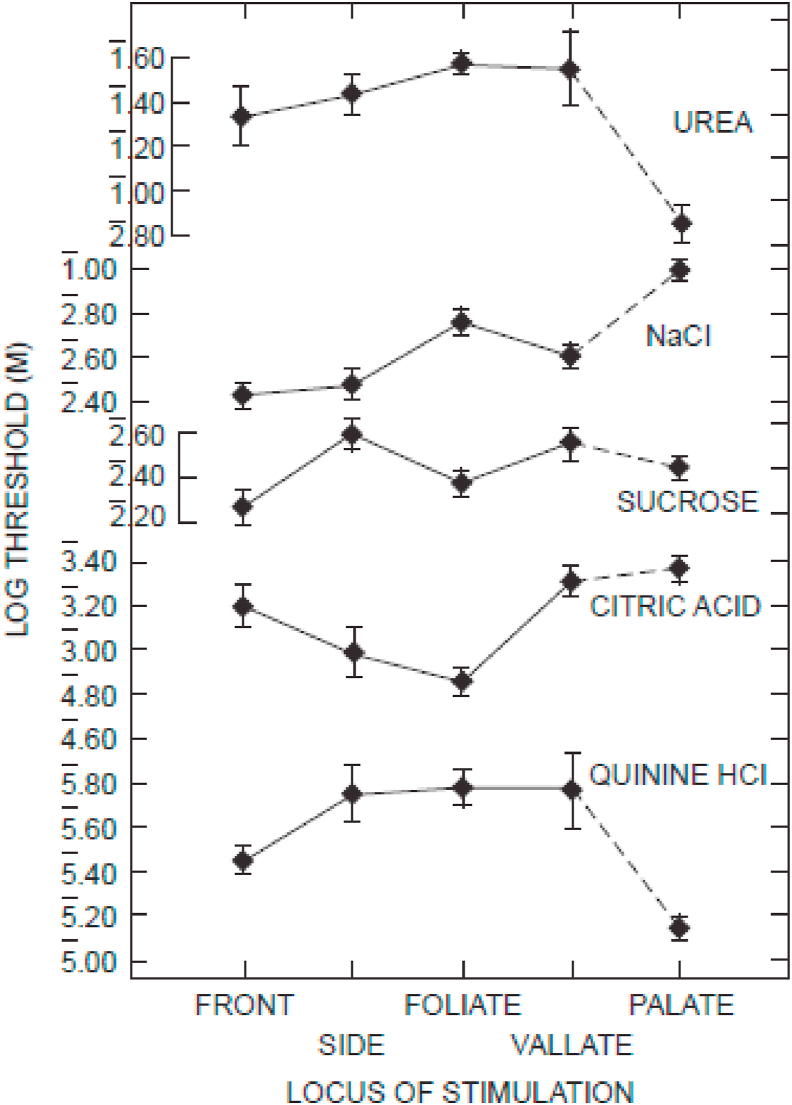
Log taste thresholds for four tongue loci and the soft palate, for urea, sodium chloride, sucrose, citric acid, and quinine hydrochloride. The horizontal lines indicate ±1 standard error of the mean. Quinine is bitter, sucrose is table sugar, citric acid is vitamin C (sour), and urea tastes like ammonia. The y-axis indicates the lowest concentration (threshold) that participants were able to taste. [Reprinted from Collings (1974, Fig. 1) with permission.].

One of the most systematic studies of the human responsiveness to basic tastes across different locations within the oral cavity where taste receptors have been documented was reported by Collings (1974). In Collings’ Experiment 1, thresholds for stimuli designed to elicit each of the basic tastes, including NaCl, sucrose, QHCl quinine (bitter), urea (bitter and sour), and citric acid were assessed in three groups of five participants. Each group of participants was exposed to four of the five tastants on the tongue, palate, or on both locations. Collings applied the tastants to the front and side of the tongue (fungiform papillae), to foliate and vallate papillae, as well as to the palate. Her results highlighted significant threshold differences as a function of the skin site stimulated. In particular, the thresholds for detecting acidity were significantly lower on foliate and fungiform (side) papillae; The threshold for sweetness was found to be lower on the front of the tongue than on the side (presumably both involving the stimulation of fungiform papillae); The threshold for detecting salt increased somewhat from front to back of the tongue/mouth (cf. Green and George, 2004; Matsuda and Doty, 1995); Meanwhile, bitter thresholds were actually lower for the fungiform papillae on the front of the tongue and for the soft palate than for the vallate papillae (see Fig. 4).

Collings (1974) and Nilsson (1977, 1979), using recognition threshold measures, confirmed Hanig's (1901) results for sweet-, salty-, and sour-tasting compounds. However, Collings reported that sensitivity to bitter compounds to be greatest on the tip, while Nilsson observed such sensitivity to be greatest on either the sides or the back of the tongue, depending upon the particular participant. In terms of the variations in taste thresholds across the surface of the tongue, Collings' (1974) and Hanig's (1901) data agree inasmuch as both studies highlight threshold differences for the basic tastes around the perimeter of the tongue, but these differences are much smaller in magnitude, and seemingly inconsistent across studies, than Boring's (1942) summary may inadvertently have led those authors writing the textbooks to believe (Bartoshuk, 1993).

Sato et al. (2002) examined gustatory thresholds for the four basic tastes on the tongues of smokers and non-smokers, assessing responses on the centre of the fungiform papillae, foliate papillae, and soft palate. Meanwhile, Doty et al. (2016) assessed sensitivity to three tastants, sucrose, sodium chloride, and caffeine on 16 regions on tongue surface using a criterion-free signal detection method in young middle-aged and older participants. Their results highlighted the existence of significant posterior to anterior, and medial to lateral, gradients of increasing performance.

### 3.2 Anterior-posterior differences in the suprathreshold range

**Fig. 5:**
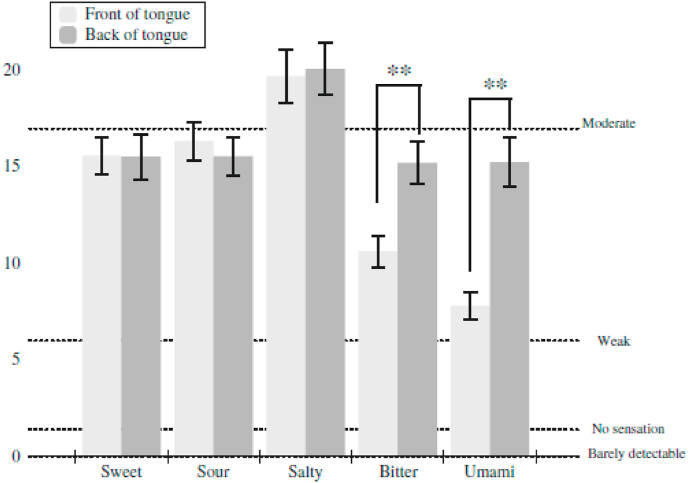
Mean intensity ratings on the front and the back of the tongue for sweet (2.0 M sucrose), sour (112 mM citric acid), salty (1.12 M NaCl), bitter (2 mM quinine), and umami (200 mM monosodium glutamate and 100 mM inosine monophosphate). Dotted lines show the relative positions of the labelled descriptors on the gLMS. Significantly different mean values (p < 0.001) between the front and back areas for a taste are denoted with ** [Reprinted with permission from Feeney and Hayes (2014, Fig. 3).].

In Collings’ (1974, Experiment 2, N = 20 participants) study, the slope of the suprathreshold intensity (or magnitude) function relating taste magnitude to the concentration of the solution was found to vary with the locus of stimulation for all compounds tested. The functions for the majority of the compounds were steepest at the vallate and foliate loci (see also Hara, 1955; Mattes, 1988; McBurney, 1969; Smith, 1971). Feeney and Hayes (2014) conducted a study in which they presented the five basic tastes to the front and back of the tongue and compared *suprathreshold* taste intensity ratings. Intriguingly, both bitter and umami stimuli were rated as significantly more intense when presented on the back of the tongue (confirming earlier claims regarding umami from Yamaguchi and Ninomiya, 1999), while no difference was reported for sweet, sour, or salty taste solutions (see Fig. 5).

According to a study on ten participants reported by Sandick and Cardello (1981; Experiment 2), significantly greater sour responses were reported in response to citric acid and NaCl in circumvallate papillae while significantly greater salty responses to these compounds were reported on the anterior tongue. These results led the researchers to argue that differentiating between the tastes of salts and acids may depend on a comparison of the input from both parts of the tongue. Green and Hayes (2003) reported that the bitter taste associated with capsaicin also varies as a function of tongue location stimulated, being significantly greater at the back of the tongue.

**Fig. 6:**
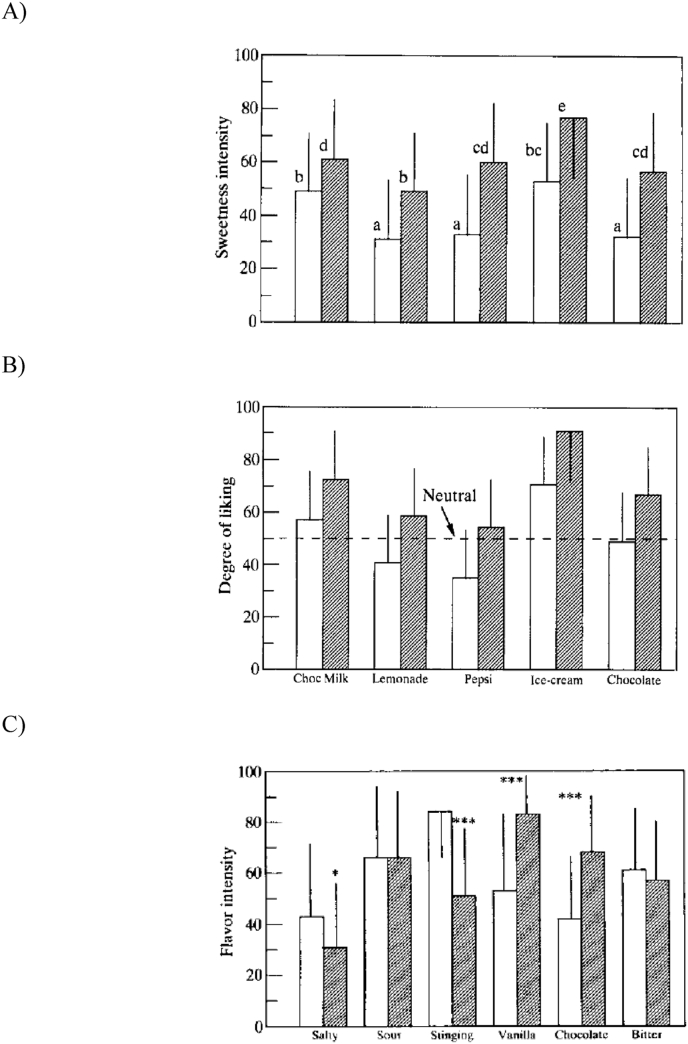
Results of a study by Hyde, presented in Hyde and Witherly (1993), in which participants rated the taste (sweetness intensity; A) and liking (B) of five everyday foods on dipping their tongue in/on the food and when swallowing. C) Location differences for other taste/flavour qualities. Bars with the same letter (in panel A) were not significantly different. Each beverage in panel B differed significantly by tongue locus. Open bars – tongue dip; Filled bars – swallow. [Asterisks in C) indicate significant difference in flavour intensity between tongue dip and swallowing.] [Reprinted from Hyde and Witherly (1993), with permission.].

Hyde and Witherly (1993) presented commercial sweet beverages, bittersweet pieces of chocolate, and ice-cream as the experimental stimuli to the front and back of their participants’ tongues. Their results highlighted anterior-to-posterior differences in perceived intensity and liking, with the various foods being rated as sweeter on swallowing than when the tip of the tongue was dipped in the food (see Fig. 6). Given that sweet stimuli are innately pleasurable (Bartoshuk and Klee, 2013), this might perhaps be considered as constituting a hedonic gradient along the anterior-posterior axis.^3^ These results led Hyde and Witherly to conclude that stimulation of the circumvallate papillae evokes stronger perceived sweetness intensities than does stimulation of just the fungiform papillae on the tip of the tongue.

### 3.3 Subjective localization of taste stimuli: ‘Tactile capture’

The experienced location of gustatory stimuli can be ventriloquized to the location of tasteless tactile stimuli. Todrank and Bartoshuk (1991) demonstrated that people's experience of the perceived source of an in-mouth tastant tends to follow the oral-tactile stimulation that they can feel moving across their tongue. In particular, gustatory stimuli presented from a fixed location on the tongue were perceived to follow a tasteless tactile probe (i.e., a Q-tip) that was moved slowly across the participant's tongue by the experimenter (see also Green, 1991, 2002; Lim and Green, 2008). Such ‘tactile capture’ even occurs when the gustatory and tactile stimuli are presented from quite different positions on the tongue (cf. Caclin et al., 2002; Jackson, 1953).

According to Linda Bartoshuk, gustatory sensations are not inherently localizable but instead their localization depends on accompanying somatosensory cues (Miller and Bartoshuk, 1991; Todrank and Bartoshuk, 1991). Nevertheless, it has been demonstrated that participants are able to determine the side of the tongue on which a pure tastants is presented (e.g., von Békésy, 1964; Shikata et al., 2000; Von Skramlik, 1924) thus showing some spatial discriminative ability. Meanwhile, Delwiche et al. (2000) conducted an intriguing study demonstrating that their participants were able to identify a tasty target stimulus from in amongst a number of tasteless distractor stimuli. Such results highlight how the perceived localization of taste stimuli often/typically results from the multisensory integration of gustatory cues with tactile cues (e.g., Calvert et al., 2004; Simon et al., 2006; Spence, 2015b). Furthermore, according to Shikata et al. (2000, p. 693): *“the mechanisms of taste localization are not known. We have three hypotheses for the mechanisms of taste localization: (I) an oral-lingual gustotopic map in the brain, (2) taste-induced tactile perception, and (3) polymodal taste fibers that are also sensitive to touch and may join into the somatotopic sensory maps.”* (cf. Cerf-Ducastel et al., 2001; Peng et al., 2015; Robinson, 1988).

One of the open questions in this area concerns whether tactile stimulation elsewhere in the oral cavity can also capture the subjective localization of taste perception. So, for example, one might wonder whether tactile stimulation on the surface of the tongue would lead to the capture of gustatory stimuli presented on the soft palate, or vice versa. However, I am unaware of any research relevant to these questions. There are undoubtedly limits to such tactile capture of taste as generally it does not seem possible to ventriloquize taste sensation outside the oral cavity (though see Michel et al., 2014, for one specific exception).

### 3.4 Astringency

Astringent stimuli, which according to the North American winemaker Clark Smith are typically experienced asymmetrically on the tongue (Smith, 2014) may presumably also lead to the illusory spatial localization of the associated taste properties (see also Green, 1993). According to the definition of American Society for Testing and Materials, astringency refers to *“the complex of sensations due to shrinking, drawing or puckering of the epithelium as a result of exposure to substances such as alums or tannins”* (American Society for Testing and Materials (ASTM), 2004). Many astringent stimuli are associated with bitter taste sensations, such as, for example, the phenolic compounds in young red wines (e.g., Brossaud et al., 2001; Ma et al., 2014; Robichaud and Noble, 1990; Shore, 1892, p. 210). That said, while the research suggests that astringency may be a trigeminally-mediated sensation (Jiang et al., 2014), it may be that different astringent stimuli are experienced somewhat differently (Lee et al., 2012).

Smith (2014, pp. 104–105) makes much of the different locations in the oral cavity where different kinds of astringency are experienced. He localizes minerality to the back of the mouth (further back than acidity with which it is apparently often confused). A little later in his book on *Postmodern winemaking*, Smith suggests that the different kinds of tannins – green, dry, melted, hard, and parching/numbing (oak) can be distinguished, in part, by the different regions of the oral cavity they are experienced as originating from. There is, however, an absence of peer-reviewed evidence on this point.

Astringency, which has been considered to be a tactile sensation (Breslin et al., 1993), is typically experienced in the middle posterior part of the tongue. In such cases, it is easy to imagine how bitterness may well be localized to the same part of the tongue, as a result of somatosensory ventriloquism (cf. Todrank and Bartoshuk, 1991). To the extent that bitter astringency is far more common than are astringent sensations that are paired with any of the other basic tastes (with the possible exception of sour; see Lea and Arnold, 1978), this may perhaps help to explain the widespread experience of bitter sensations seemingly being localized to the back of the tongue (Bajec and Pickering, 2008a; Lea and Arnold, 1978; Ishikawa and Noble, 1995; Peleg et al., 1999). Consistent with such a claim, Lim and Green (2008) have suggested that (weak) bitter tastants may be especially prone to tactile capture in the oral cavity.

One other factor that may be relevant to consider here relates to the spatial distribution of the salivary ducts, since the presence/composition of saliva can influence taste/flavour perception (see Ma et al., 2014; Running, 2018; Schwartz et al., 2021; Spence, 2011). Not only are the salivary ducts asymmetrically distributed across the surface of the tongue, the composition of saliva also differs somewhat between the different classes of salivary duct. Salivary proteins are thought to play an important role in the perception of oral astringency, which, as we have just seen is localized subjectively to the back of the tongue. Running (2018, p. 236) also notes how: *“minor glands in the posterior of the tongue (von Ebner's glands) secrete directly into the clefts of the circumvallate and foliate papillae, where the densest population of taste buds in the mouth are located.”* Running summarizes the emerging evidence concerning how saliva modifies the perception of sourness, saltiness, bitterness, and oleogustus. Salts and sugars are thought to be somewhat more soluble in saliva than are the stimuli giving rise to the other basic tastes (cf. Canon et al., 2018; Canon and Neyraud, 2017; Tiwari, 2011). It is therefore interesting to speculate on whether this might link to the perceived localization of salty and sweet taste sensations to the periphery of the tongue, where there is likely to be more saliva. One issue that potentially follows on from this is then to note how, in some people, salivation can be induced by food extrinsic cues, such as the sight of a lemon being cut (see Spence, 2011, for a review; cf. Proserpio et al., 2017).

### 3.5 Irritant and pungent trigeminal stimuli

Certain irritant and pungent trigeminal stimuli are also experienced asymmetrically in the oral cavity and throat. For instance, swallowing solutions of capsaicin has been reported to lead to more pronounced irritation in the throat than in the oral cavity, while for piperine, another pungent stimulus, the sensation on swallowing is equally intense in both the throat and oral cavity (Rentmeister-Bryant and Green, 1997). Trigeminal stimuli also elicit irritation, albeit of reduced intensity, when presented to the lip (Lawless and Stevens, 1988). The qualitative similarity of bitter taste and burning oral sensations (Lim and Green, 2007), again perhaps leading to increased capture by trigeminal stimulation. Intriguingly, the pungent sensory responses to oleocanthal or ibuprofen are primarily located in the throat (Peyrot des Gachons et al., 2011). In such cases, the nonspecialized nerve endings in the epithelium giving rise to such pungent responses are expressed in the throat but not in the oral cavity. Intriguing research by Peyrot des Gachons and her colleagues has demonstrated that this most unusual pattern of irritation is a consequence of both the specificity of oleocanthal for a single sensory receptor and the fact that this sensory receptor is anatomically restricted to the pharynx within the oral cavity. *“These observations suggest a poor expression of the OC receptor on the trigeminal fibers innervating the human anterior tongue compared with the human pharyngeal and nasal nerve afferents.”* (Peyrot des Gachons et al., 2011, pp. 1004–1005). Note that ibuprofen has been documented to show the same idiosyncratic spatial pattern of responding (Breslin et al., 2001).

Many bitter-tasting toxins are perceived more strongly in the posterior oral cavity than the anterior (Danilova and Hellekant, 2003). Recording from the chorda tympani and glossopharyngeal nerves in mice, the latter researchers noted that the responses from the two nerves were not the same. In general, sweeteners tended to give rise to larger responses in the chorda tympani than in the glossopharyngeal nerve, while responses to bitter taste in the glossopharyngeal nerve were larger (see also Green and Schullery, 2003; Hellekant et al., 1997; Ninomiya et al., 2000). Meanwhile, according to Pfaffmann et al. (1967, p. 361), quinine is a much less effective stimulus for the nerve fibers innervating the fungiform papillae (chorda tympani) than for those innervating the circumvallate and foliate papillae of the posterior third of the tongue (glossopharyngeal nerve). Higgins and Hayes (2019) have also reported some intriguing perceptual differences in regional patterns of stimulation when comparing hops extract to quinine.

### 3.6 On the role of active tongue movements when tasting

The everyday mastication/consumption of food products typically involves active tongue movements, and their role in contributing to everyday tasting and multisensory flavour perception should not be ignored (Colvin et al., 2018; Hyde and Witherly, 1993; Spence, 2019). Such intentional movements may well require/capture a taster's attention, and hence result in the localization of gustatory stimuli to the actively-moving tongue, rather than to other taste receptors (e.g., in the soft palate) that are stimulated passively at around the same time (cf. Krakauer and Dallenbach, 1937; Meiselman et al., 1972). Colvin et al. (2018) reported that when tastants were presented passively on the tongue, the front surface was more responsive to sucrose and maltooligosaccharides while no regional differences were observed for quinine and monopotassium glutamate. By contrast, when the participants were encouraged to actively taste, the posterior tongue was more responsive to quinine and monopotassium glutamate, while no differences were observed for sucrose or maltooligosaccharides. These results highlight how regional differences in responsiveness to different tastants may depend on the mode of tasting – active vs. passive. Note that such naturalistic tongue movements (active tasting), which tend to constitute a ubiquitous feature of our everyday consumption of food and drink, are typically absent from the design of highly-controlled psychophysical studies of taste perception where passive presentation is the norm.

### 3.7 On the role of active tongue movements when tasting

It is interesting to speculate as to whether such spatial differences in taste perception, as a function of the part of the surface of the oral cavity that is stimulated, are a regular feature of our gustatory experiences. Here, though, it is worth noting how the set-up of traditional taste psychophysics experiments tends to be very different from the naturalistic conditions of everyday food consumption. Given that consumers appear to have a prior to assume that everyday foods will present a uniform taste (e.g., Woods et al., 2010), one might wonder whether some sort of perceptual constancy may operate in the ‘world of taste’ (cf. Bartoshuk, 1980; Blakeslee and Fox, 1932) as has previously been suggested to operate in the case of orthonasal olfaction (Teghtsoonian et al., 1978). Perceptual constancy refers to the idea that what the brain really wants to know about is the nature of the distal stimulus, and so sometimes discounts marked variations in the nature of the proximal stimulus. Such mechanisms, should they exist for taste perception in the oral cavity, are presumably much more likely to operate in the case of familiar foods (e.g., as used in Hyde and Witherly, 1993, study), and under naturalistic tasting conditions (i.e., when the various taste buds situated throughout the oral cavity are likely to be stimulated simultaneously). This is because our memory of the taste/flavour (what might be called the distal stimulus) is likely to play a much more important role in the taste/flavour experience (the proximal stimulus) of familiar foods, whereas memories are, by definition, less relevant in the case of those foods that happen to be unfamiliar. Ultimately, what the brain is presumably trying to ascertain is the nutritional qualities of the food itself, regardless of the specific oral sensations that happen to be associated with that stimulus (Breslin, 2013).

### 3.8 On the spatial constraints on thermal tasting

Another spatially-localized form of taste perception that should be mentioned here concerns thermal taste (e.g., Botha et al., 2021; Skinner et al., 2018). A subset of individuals perceive a taste when the tongue is rapidly rewarmed after having been cooled (Cruz and Green, 2000). Research on such ‘thermal tasters’ (e.g., Bajec and Pickering, 2008b; Green and George, 2004), has revealed that thermal sweetness is the most common of the thermally-induced tastes, and is typically experienced on the tip of the tongue. Thermal tastes are perceived by ∼50% of individuals when the tongue is rapidly re-warmed after being briefly cooled to 15–20 °C. While the mechanism underlying thermal taste is unknown, it has been hypothesized that it may result from a temperature-sensitive process related to chemical taste transduction (Cruz and Green, 2000). Whatever the most appropriate explanation turns out to be, the existence of thermal tasting highlights the fact that gustatory qualities can be elicited by thermal stimuli rather than necessarily signalling the presence of the respective tastants. Perhaps more importantly, only certain taste qualities can be induced thermally, and those that can tend to be spatially localized to specific parts of the tongue: For instance, a sweet taste is typically induced on the front and a sour taste response on the side of the tongue.

### 3.9 Subjective localization of taste stimuli: Beliefs about the localization of taste

Finally, in this section, it is perhaps worth considering whether people's beliefs about the localization of particular taste qualities within the oral cavity may also influence their spatial perception of gustatory stimuli. The participants in a study by Crisinel et al. (2012) rated the perceived location on the tongue of the taste sensation associated with tasting a sample of bittersweet cinder toffee while exposed to sonic seasoning that was putatively either sweet or bitter. That said, the perceived location of the taste sensation shifted slightly, albeit not significantly, toward the front of the tongue with sweet music (when the toffee was rated as tasting sweeter) and toward the rear of the tongue when listening to the bitter music (when the toffee was rated as tasting more bitter). Even in this small study, increased bitter ratings were significantly correlated with participants localizing the taste/flavour experience further toward the back of their mouths. Such results might be taken to suggest that people implicitly associate sweet with the tip of the tongue and bitterness with the posterior tongue. Consider here only how such a belief (or association based on previous experience) might, in turn, influence the perceived localization of gustatory stimuli. Indeed, elsewhere in psychology, mentally imagined visual stimuli have been shown to lead to the mislocalization, or ventriloquism of paired auditory stimuli (cf. Berger and Ehrsson, 2013). Furthermore, subjective ratings of, and neural responses to, basic tastants have also been shown to be modulated by expectation (e.g., Nitschke et al., 2006; Wilton et al., 2018; Woods et al., 2011). Selective attention has also been shown to modulate the detectability of weak taste stimuli (Marks and Wheeler, 1998), while food expectations can modulate salivation (Keesman et al., 2016; Spence, 2011).

### 3.10 Individual differences in the spatial distribution of taste receptors

The perceived intensity of taste typically aligns with the number of receptors that are stimulated, though the sometimes subtle differences in taste intensity reported by supertasters not obviously appearing to match the 14-fold variation in the number of taste buds that have been reported to exist in fungiform papillae between individuals at either end of the supertaster spectrum (Bartoshuk, 1993; Miller et al., 1990a, 1990b; Reedy, cf. Zuniga, Davis, Englehardt, Miller, Schiffman and Phillips, 1993; see also Delwiche et al., 2001; Oakley, 1985). According to Bartoshuk (2000), supertasters have the largest number of taste buds, nontasters the smallest. The differences in the number of receptors are very large. For example, the average number of taste buds per square centimetre was 96, 184, and 425 for nontasters, medium tasters and supertasters, respectively. These measures were taken on the anterior tongue (thus targeting fungiform papillae; see also Tepper and Nurse, 1997). I am not aware of any suggestion that there is a similar variation in the density of taste buds for foliate/vallate papillae (cf. Bartoshuk et al., 1996). At the same time, however, Garneau et al. (2014) have provided evidence to suggest that there is actually no relation between the density of fungiform papillae and an individual's taster status. Note that the distribution of taste receptors, rather than simply the gross morphology (i.e., papillae density) would be more relevant here. However, the phenomenon of spatial summation should also be considered (Linschoten and Kroeze, 1991; Smith, 1971).

## 4 On the relation between taste receptors and multisensory flavour experiences

As Harper et al. (1966, p. 325) noted long ago: *“The difficulty of relating psychophysics and physiology is especially great for the qualitative aspect of gustation, since in this system very little is known about the relevant physical variables of the stimuli”*. Indeed, a quick consideration soon reveals that there is often a substantial disconnect between the spatial distribution of taste receptors, and taste perception in the context of multisensory flavour experiences, given that we never really consume pure tastants (see Spence et al., 2015). For one thing, consider here only how the taste buds are relatively widely separated (Jung et al., 2004), and yet we experience the taste qualities of food as if they are present at all points across the tongue (i.e., without any gaps). According to Bartoshuk (1993), such a phenomenon can be considered as a kind of gustatory ‘filling-in’; filling-in, note, is a phenomenon that has been studied extensively in vision at the blind spot (e.g., Pessoa and De Weerd, 2003). Something similar also happens on the skin surface with thermal receptors being widely separated, and yet a continuous thermal feeling is typically experienced on the skin surface (see Gallace and Spence, 2014; though see also Green and Cruz, 1998, on the existence of ‘warmth insensitive fields’ on the skin surface). However, to date there has been rather less interest in the case of gustation.

### 4.1 Taste experiences not triggered by tastants

It is important to recognize that our experience of particular taste qualities (such as sweet or bitter) is not determined solely by the activation of the relevant gustatory receptors for sweetness and bitterness by an appropriate tastant. As we have seen already, specific taste percepts can, at least under a subset of conditions, be elicited by means of thermal cues; see Section 3.5). On the one hand, gustatory stimuli can sometimes be elicited by food aromas that have frequently been paired with a particular tastant in the past (Stevenson and Boakes, 2004). Indeed, under certain conditions, taste percepts can be induced by the appropriate olfactory stimulus in the absence of the relevant gustatory input. And, should a tastant be present, then the addition of the relevant (i.e., congruent) aroma may well lead to the modulation (i.e., enhancement) of taste intensity (see Spence, 2015a, 2022, for reviews).

However, the question of where such olfactorily-induced taste enhancement effects are experienced as originating (spatially) has not, at least as far as I am aware, been studied empirically. It is easy to imagine, though, how there might be a link to the phenomenon of oral referral (see Spence, 2016, for a review). Should this be the case, then the congruency of the taste-odour combination, and/or the intensity, or attention-capturing quality, of the gustatory stimulus might be expected to determine where exactly the olfactory stimuli, and thus perhaps also the associated taste property, are localized subjectively. However, further research will be needed to establish whether this is indeed the case.

## 5 Conclusions

The tongue map (e.g., Wiese, 2000), whose popularity during the middle decades of the 20th Century, has been attributed to the publication of Edwin Boring's (1942) psychology textbook (see Bartoshuk, 1993), has long been dismissed by psychophysicists (e.g., Bartoshuk, 1993), neuroscientists (Chandrashekar et al., 2006), and journalists (O'Connor, 2008) alike. However, as highlighted by the present review, there is quite some ambiguity in the literature concerning what exactly the claim being made by those who supported the existence of the tongue map, actually was. Ultimately, the status of the tongue map depends on the specific claim about the spatial properties of taste that is being made. For, if the claim is taken to refer to the spatial distribution of receptors (for sweet, bitter, salty, and sour), the neuroscience research that has been published in recent decades has unequivocally demonstrated that the sensory receptors for the different taste qualities are indeed to be found across the tongue (Chandrashekar et al., 2006; Chen et al., 2011; Li et al., 2002; Yarmolinsky et al., 2009), and hence no further consideration need be given to such a segregated taste receptor account.^4^

If, however, the claim is instead taken to relate to the perceived spatial localization of taste qualities, and/or about sensitivity differences on different parts of the tongue, then psychophysics is more likely to provide a meaningful answer than neurophysiology. Indeed, it is worth remembering that Hanig (1901) original work was titled ‘The psychophysics of taste’. Though according to Bartoshuk (1993, p. 22), Hanig: *“believed that if the thresholds for his four stimuli (sucrose salt, quinine sulfate, and hydrochloric acid) could be shown to vary differentially around the perimeter of the tongue, then this would* *support* *the argument that these four tastes had distinct physiological mechanisms.”* What is more, numerous studies published over the last half century or so have highlighted sensory-discriminative differences across the tongue as well as elsewhere in the oral cavity where taste receptor cells have been documented (e.g., Collings, 1974; Feeney and Hayes, 2014), thus supporting Hänig's early findings.

For those who interpret the tongue map as highlighting differences in sensitivity, the relevant question to ask then becomes one of whether the spatial differences in sensitivity are sufficiently great to merit consideration or not. While different researchers have come to different conclusions to this question (e.g., see the quote from Munger in Hammond, 2017, mentioned earlier), it might anyway be argued that threshold measures are actually of rather less relevance than any spatial differences in people's responses to suprathreshold taste stimuli (see Feeney and Hayes, 2014; Hyde and Witherly, 1993). Ultimately, one of the important points to stress here is that taste localization, just like other aspects of flavour perception, is a fundamentally multisensory phenomenon (Maier and Elliott, 2020; Simon et al., 2006; Spence et al., 2015).

The psychophysical evidence clearly highlights the fact that there are differences in gustatory sensitivity for tastants applied to the different sites where taste receptors have been documented such as the tongue, palate, and pharynx (Collings, 1974; Hanig, 1901; Henkin and Christiansen, 1967; Nilsson, 1977, 1979; Shore, 1892). Significant differences in people's response to suprathreshold taste stimuli have also been reported between the anterior and posterior tongue (e.g., Doty et al., 2016; Feeney and Hayes, 2014; Hyde and Witherly, 1993; Sandick and Cardello, 1981; Sato et al., 2002; see also Halpern and Nelson, 1965), as well as along the medial to lateral axis (Doty et al., 2016). Feeney and Hayes (2014, p. 147) write that: “Although all taste sensations were experienced all over the tongue, once again disproving the mythical tongue map, we also observed bitter and umami taste perceptions to be significantly greater on the posterior tongue than the anterior tongue.”

A number of possible explanations for the apparent mismatch between the supposedly uniform spatial distribution of different taste receptor cells on the tongue (Chandrashekar et al., 2006; Yarmolinsky et al., 2009), and the spatial qualities of taste experiences have been put forward. They include an anterior-posterior gradient in food acceptance/avoidance, and the tactile, or oral-somatosensory, ventriloquism of taste sensations (e.g., Green, 2002; Lim and Green, 2008; Todrank and Bartoshuk, 1991), possibly linked to both gustatory and thermal/tactile inputs being conveyed by the glossopharyngeal nerve. Ventriloquism here refers to the idea that the apparent spatial source of taste is mislocalized, or ventriloquized, to the location of any simultaneous tactile stimulation on the tongue (Todrank and Bartoshuk, 1991). Certain pungent and trigeminal stimuli, such as oleocanthal, ibuprofen and capsaicin are sensed more strongly in the throat than in the oral cavity (Breslin et al., 2001; Rentmeister-Bryant and Green, 1997; Peyrot des Gachons et al., 2011). Expectancy effects, and olfactorily-induced taste enhancement should not be ignored either (Spence, 2022), nor, for that matter, should the role of active tongue movements and swallowing under conditions of naturalistic consumption (cf. Colvin et al., 2018; Running and Hayes, 2017).

Perhaps the larger point to bear in mind here, though, is that in order to try and understand the fundamental mechanisms underlying gustatory perception, traditional taste psychophysics has typically tended to consider local, targeted, passive stimulation by isolated gustatory stimuli rather than the situation of whole mouth active stimulation by a range of different chemosensory stimuli as is normally the case during everyday tasting and food consumption (see Colvin et al., 2018; see also Delwiche et al., 2000; Collings et al., 1976). Here, one might onsider the flow system designed for taste research (e.g., Pfaffmann, 1935; see also Ashkenazi et al., 2004; McBurney, 1969).

Numerous psychophysical studies conducted over the last half century or so have, then, demonstrated a spatial modulation of the sensory-discriminative (and possibly also hedonic; Hyde and Witherly, 1993) responses to taste experiences involving gustatory stimuli presented in both the threshold and supra-threshold range. Acquiring a better understanding of the spatial distribution of gustatory receptors, and the link to the associated perceptual qualities,^5^ especially under conditions of naturalistic food consumption, is likely going to become increasingly important in the years ahead, as food scientists and product developers consider how to optimize the taste of new food products (see Spence, 2021, for a review). Targeting the delivery of tastants to those parts of the oral cavity where taste-receptor cells contributing most to the perceived taste of foods may ultimately enable the development of commercial processed food products less of the more unhealthy ingredients such as sugar, salt, and fat. Or, as Booth (1990) put it a little over three decades ago, a need exists for *“establishing what actually is in awareness that has a causal role (in ingestive behaviour)”*. However, as has just been mentioned, the most relevant findings are likely going to emerge from those studies involving naturalistic food stimuli and consumption behaviours rather than the passive delivery of isolated tastants as has been such a popular feature of traditional taste psychophysics research in previous decades (cf. Cattaneo et al., 2020).

At the same time, however, there has also been growing interest in the relationship between the composition of oral microbiota and taste perception (Cattaneo et al., 2019). It currently remains an intriguing yet open question as to whether there might also be a relation between gut health and the spatial aspects of taste perception (though see Dutt et al., 2021, for some intriguing recent data in this regard).

## CRediT authorship contribution statement

**Charles Spence:** All parts of the manuscript were, Writing – original draft.

## Declaration of competing interest

The author declares that he has no known competing financial interests or personal relationships that could have appeared to influence the work reported in this paper.

## Footnotes

1 As Bartoshuk (1993, p. 21) puts it: *“Understanding human taste requires that we appreciate the fact that the taste apparatus is spatially distributed. There is variation in the number and spacing of taste buds and the nerves innervating these taste buds interact in complex ways that were not suspected a few years ago. Both of these new anatomical observations have important consequences for our taste worlds.”*

2 I would argue that this is not quite what they mean, as these researchers themselves highlight the fact that taste receptor cells are only found on certain parts of the upper surface of the tongue (see Chandrashekar et al., 2006, Fig. 1).

3 This might perhaps be something of an oversimplification, given that those who appreciate beer might well argue that they prefer the particular bitter sensation that they experience on the back of the tongue.

4 As Breslin (2013, p. R409) puts it: *“Whereas it was once hypothesized that these receptors should be expressed in particular zones according to presumed taste quality regions of the mouth, we now believe that the receptor expression zones are heavily overlapping in most regions of the mouth.”*

5 According to Breslin (2013), the relevant attributes of a taste percept include its quality, intensity, hedonics, spatial localization, and temporal dynamics.
